# Skewed X-Chromosome Inactivation and Parental Gonadal Mosaicism Are Implicated in X-Linked Recessive Female Hemophilia Patients

**DOI:** 10.3390/diagnostics12102267

**Published:** 2022-09-20

**Authors:** Ming-Ching Shen, Shun-Ping Chang, Dong-Jay Lee, Wen-Hsiang Lin, Ming Chen, Gwo-Chin Ma

**Affiliations:** 1 Division of Hematology-Oncology, Department of Internal Medicine, Changhua Christian Hospital, Changhua 50046, Taiwan; 2 Hemophilia Treatment and Thrombosis Center, Changhua Christian Hospital, Changhua 50046, Taiwan; 3 Department of Genomic Medicine and Center for Medical Genetics, Changhua Christian Hospital, Changhua 50046, Taiwan; 4 Welgene Biotechnology Company, Nangang Business Park, Taipei 11503, Taiwan; 5 Department of Obstetrics and Gynecology, Changhua Christian Hospital, Changhua 50046, Taiwan; 6 Department of Obstetrics and Gynecology, College of Medicine, National Taiwan University, Taipei 10617, Taiwan; 7 Department of Medical Genetics, National Taiwan University Hospital, Taipei 10617, Taiwan; 8 Department of Medical Sciences, National Tsing Hua University, Hsinchu 300044, Taiwan; 9 Department of Post-Baccalaureate Medicine, College of Medicine, National Chung Hsing University, Taichung 40227, Taiwan; 10 Department of Medical Laboratory Science and Biotechnology, Central Taiwan University of Science and Technology, Taichung 406053, Taiwan

**Keywords:** female hemophilia, X-linked, heterozygous, inactivation, skewed XCI, gonadal mosaicism

## Abstract

Background: Hemophilia A (HA) and B (HB) are X-linked recessive disorders that mainly affect males born from a mother carrier. Females are rarely affected but a number of mechanisms have been suggested in symptomatic females, such as skewed X-chromosome inactivation (XCI), chromosomal rearrangements, and hermaphrodites. Different methodologies are required to elucidate the underlying causes of such diseases in female patients. Methods: Three families with female hemophilia patients, including two HA and one HB, were enrolled for genetic analyses. Cytogenetics, molecular examinations on *F8* and *F9* genes, XCI assay, and linkage analysis were performed. Results: All three female patients are demonstrated to be heterozygous for an *F8, or F9* mutation: one patient is inherited from her unaffected mother and the other two are sporadic cases. All three patients exhibit skewed XCI. The inherited patient is found to be unmethylated in the maternal X chromosome, which increases the potential for the expression of the mutant allele. The two sporadic cases are hypomethylated or unmethylated in the paternal X chromosome, suggesting that paternal gonadal mosaicism may exist in these families. Conclusions: In addition to screening for coagulation function, different genetic analyses are mandatory to explore the nature of mechanisms responsible for the X-linked recessive disorders in female patients as shown in this study. Our results confirm that skewed XCI is responsible for hemophilia in heterozygous female patients. Likewise, our results implicate that parental gonadal mosaicism, followed by skewed XCI, contributes to hemophilia in “sporadic” female patients.

## 1. Introduction

Hemophilia A (HA; OMIM # 306700) and B (HB; OMIM # 306900) are X-linked recessive disorders characterized by prolonged hemorrhage following cuts, injuries, or surgeries, and in severe cases, spontaneous bleeding. The diseases are caused by a deficiency of clotting factors of the coagulation cascade (FVIII encoded by the *F8* gene for HA and FIX encoded by the *F9* gene for HB), in which the clinical severity is influenced by a decreased level of clotting factor activity (i.e., FVIII:C, or FIX:C) in plasma of the affected individuals. Severities of HA, or HB were determined based on FVIII:C, or FIX:C: severe if <1%, moderate if between 1–5% and mild if >5% and <40% of normal [1].

The X-linked hemophilias are usually identified in male children born from a nonsymptomatic carrier mother. The diagnostic evaluation is initiated by a thorough review of the patient’s personal bleeding history and family history, followed by confirmative laboratory tests, including screening tests of hemostasis, assays of clotting factor activity levels, and genetic testing. With the advance in genetic diagnosis, a heterogeneous mutational spectrum with multiple mutational events leading to hemophilia have been identified on the X chromosome, such as rearrangements involving intron 1 inversions (INV1) and intron 22 inversions (INV22), deletions, duplications, and point mutations of *F8* gene [2]. Patients with HA and HB are typically males hemizygous and females homozygous, or compound heterozygous for mutant *F8, or F9*. Heterozygous females are seldom affected because a normally expressed allele on the other X chromosome can compensate for the genetic defect, unless in conditions of skewed X-chromosome inactivation (XCI), coexistence with additional X-chromosome aberrations that cause the loss of a functional gene, and hermaphrodites, by which the wild-type allele does not express as it usual and symptoms developed [2,3,4,5,6]. We had previously reported one very rare hemophilia female patient who carries a familial deletion across the exon 1–22 of the *F8* gene on one X chromosome and possesses a de novo rearrangement (isodicentric X) on another X chromosome that leads to complete loss of a functional *F8* gene, demonstrated by multiplex ligation-dependent probe amplification (MLPA) in addition to karyotyping [2]. Therefore, for female patients, a combination of genetic analyses and an integrated approach are required to elucidate the underlying mechanism of X-linked diseases, such as hemophilias [7].

In this study, a series of genetic analyses, including chromosomal karyotyping, molecular genetic examinations, XCI analysis, and linkage analysis, were offered for three unrelated families to understand the molecular pathology of X-linked recessive HA and HB in female patients. The results demonstrated that the skewed XCI is responsible for hemophilia in females with a mutation and suggested that parental gonadal mosaicism may contribute to hemophilia in females with a *de novo* mutation.

## 2. Materials and Methods

### 2.1. Subjects

**Figure 1:**
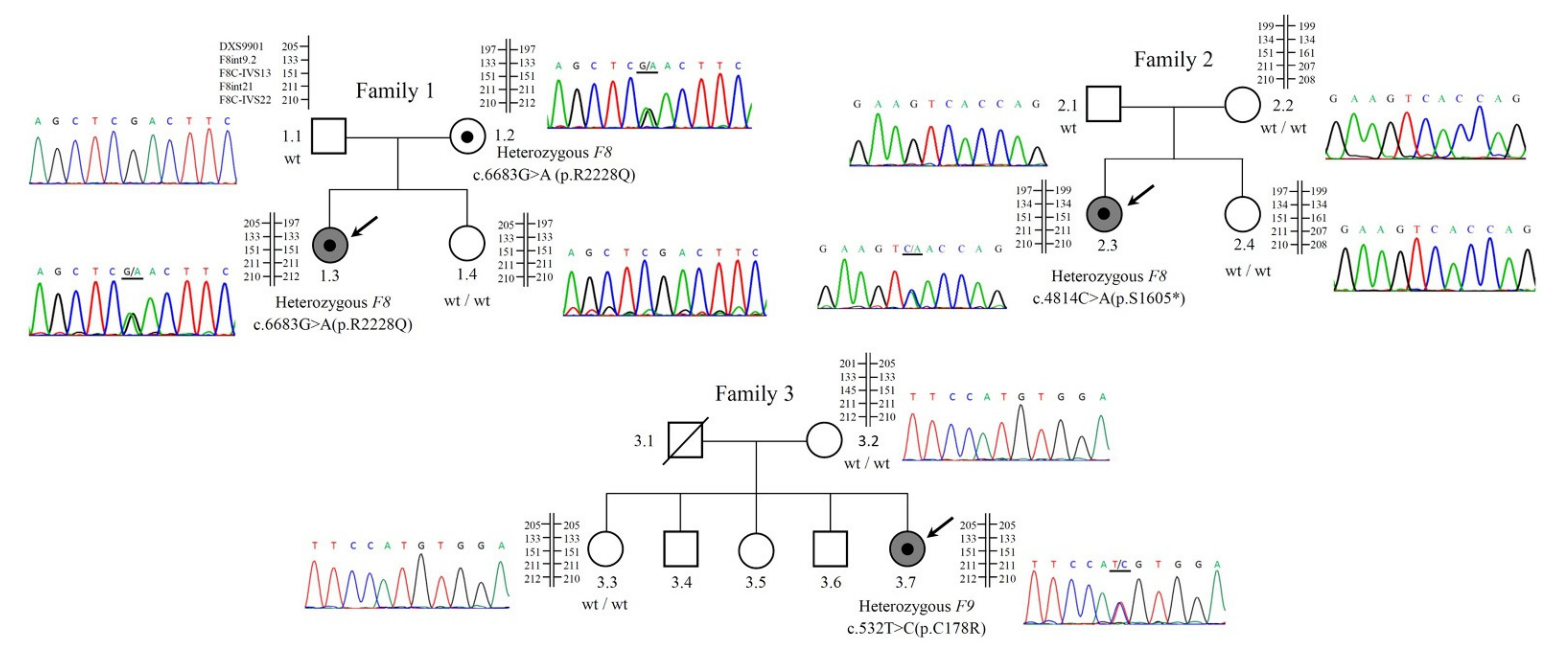
Pedigree information of three families with female hemophilia A (HA) (families 1 and 2) and hemophilia B (HB) (family 3). The female patient of family 1 (case 1.3) carries a heterozygous c.6683G>A(p.R2228Q) mutation in *F8* that was inherited from her unaffected mother (case 1.2). The female patients of family 2 (case 2.3) and family 3 (case 3.7) carry heterozygous c.4814C>A(p.S1605*) in *F8* and c.532T>C(p.C178R) mutations in *F9,* respectively; both were of de novo origin. Linkage analysis was performed by using five X-linked short tandem repeat (STR) makers (DXS9901, F8int9.2, F8C-IVS13, F8int21, and F8C-IVS22). Arrow denotes the proband. Underlined nucleotide denotes the position of the mutation. White square and circle symbol denote unaffected males and females, respectively. A white circle symbol with a black dot denote an unaffected female carrying a heterozygous mutation without skewed X-chromosome inactivation (XCI) and a gray circle symbol with a black dot denote an affected female carrying a heterozygous mutation with shewed XCI. Slash denotes an individual who is deceased. *, a nonsense mutation that changes an amino acid to a translation termination (stop) codon.

Three female patients from three unrelated and non-consanguineous families were referred to us for a differential diagnosis of hemophilia in Changhua Christian Hospital (CCH), Changhua, Taiwan. Standard coagulation testing for HA and HB diagnosis was performed for these patients (and their family members) using the methodology as previously described [8]. Briefly, the FIX:Ag was determined by ELISA with the Asserachrom IX:Ag kit (Stago UK Ltd., Theale, UK). The FVIII:C, FIX:C, vWF:Ag, and vWF:RC were examined by automated quantitative immunoassays performed on the bioMerieux VIDAS system (bioMérieux, Marcy l’Etoile, France) and ACL TOP 500 platform (Werfen, Cheshire, UK). Genotype-phenotype correlations in the three families were ascertained through the female patients (probands) (Figure 1). The clinical information of the patients and available family members was described below:

The patient of family 1 (case 1.3) is a 29-year-old female who was diagnosed of severe HA (FVIII:C < 1%, vWF:Ag 96%, and vWF:RC 100%) at 3 years old due to recurrent bleeding on puncture site. She sustained ecchymosis since the age of 6 months, followed by multiple bleeding episodes in her left knee and ankle. Her period flow of menstruation seems normal and regular. Both parents (case 1.1: FVIII:C 188%, vWF:Ag 164%, and vWF:RC 191%; case 1.2: FVIII:C 79%, vWF:Ag 141%, and vWF:RC 168%) and her younger sister (case 1.4: FVIII:C 165%, vWF:Ag 111%, and vWF:RC 106%) are healthy without blood clotting problem and were enrolled for our genetic study.

The patient of family 2 (case 2.3) is a 20-year-old female who was diagnosed of moderate HA (FVIII:C:4.6%, vWF:Ag 106%, and vWF:RC 89%) at 4 years of age due to hematoma at her forehead. Afterward, she sustained occasionally bleeding at the right knee, but a normal period amount after menarche. None of her parents (case 2.1: FVIII:C 124%, vWF:Ag 102%, and vWF:RC 108%; case 2.2: FVIII:C 70%, vWF:Ag 92%, and vWF:RC 118%) and her younger sister (case 2.4: FVIII:C 120%, vWF:Ag 115%, and vWF:RC 113%) experienced bleeding diathesis and presented normal coagulation function. Her parents and sister were also enrolled for our genetic study.

The patient of family 3 (case 3.7) is a 62-year-old female who has the diagnosis of severe HB (FIX:C < 1% and FIX:Ag < 6%) at her age of 15 years. She sustained recurrent ecchymosis followed by extensive ecchymosis on her puncture sites since her age of 2 years. Additionally, she suffered from hypermenorrhea and remarkable joint bleeding, and thus underwent total knee replacement at her right knee. Her father died without any hemophilia history when he was alive. Her mother (case 3.2: FIX:C 75% and FIX:Ag 99%) and siblings exhibited normal FIX function (case 3.3: FIX:C 100% and FIX:Ag 106%; case 3.4: FIX:C 102% and FIX:Ag 109%; case 3.5: FIX:C 78% and FIX:Ag 94%; case 3.6: FIX:C 126% and FIX:Ag 105%). Her mother and elder sister (case 3.3) were enrolled for our genetic study. All participants were followed up for cytogenetic analysis, molecular genetic examinations, XCI analysis, and linkage analysis.

### 2.2. DNA Extraction

DNA from peripheral blood cells were extracted using the PUREGENE^®^ DNA Purification Kit (Gentra Systems, Minneapolis, MN, USA) according to the supplier’s protocol. The quality and purity of DNA were evaluated based on the values and ratio of the absorbances at 260 nm and 280 nm using Nanodrop 2000 spectrophotometer (Thermo Fisher Scientific, Waltham, MA, USA).

### 2.3. Cytogenetic Analysis

Cell suspensions from peripheral blood were used for cytogenetic analysis to examined the chromosomal compositions by G-banding. Cell culture and chromosome banding analysis were performed following standard protocols [9].

### 2.4. Molecular Genetic Examinations

A combination of molecular genetic analyses was performed to screen for the *F8* and *F9* mutations. Inverse polymerase chain reaction (PCR) and multiplex PCR were performed to detect the common IVS22 and IVS1 of the *F8* gene [10,11]. Sanger sequencing was performed to detect variants on the exons and exon-intron junctions of *F8* and *F9* genes. Primer sets and PCR conditions for DNA amplification and sequencing of *F8* and *F9* genes were summarized in Tables S1 and S2. The nomenclature for the description of sequence variants follows the guidelines of the Human Genome Variation Society (https://www.hgvs.org/; accessed on 10 August 2022). Multiplex Ligation-dependent Probe Amplification (MLPA) was performed with the SALSA MLPA Probemix P178 *F8, or* P207 *F9* (MRC-Holland, Amsterdam, The Netherlands) to detect the underlying deletions that may escape detection by the PCR-based analysis due to the masking of the wild-type allele. Analysis of exon dosages by MLPA followed the guideline described in Schouten et al. (2002) [12].

### 2.5. XCI Analysis

**Figure 2:**
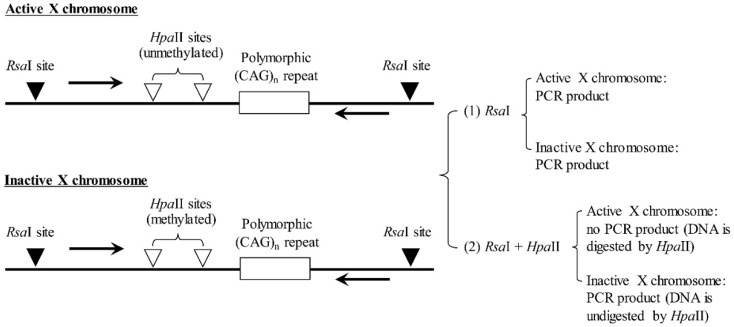
Schematic diagram of the X-chromosome inactivation (XCI) analysis of androgen receptor (AR) to distinguish between active (unmethylated) and inactive X (methylated) chromosome in females by restriction enzymes of *RsaI* (methylation insensitive) and *HpaII* (methylation sensitive), followed by polymerase chain reaction (PCR) amplification of a polymorphic (CAG)n repeat in AR. In the test of the “*RsaI*” aliquot, DNA fragments are amplified from both X chromosomes, regardless of their activity status. In the test of the “*RsaI + HpaII*” aliquot, only the DNA fragment on the inactive X chromosome is amplified. The degree of XCI was determined based on the ratio of the peak height of each AR allele in relative to the sum of the heights of both AR alleles.

The XCI patterns in female patients and their family members were examined by analyzing the methylation status of the androgen receptor (AR) as previously described [13], with minor modifications (Figure 2). In short, two aliquots of ~200 ng DNA from each case were digested with restriction enzymes at 37 °C for 16 h: one with *Rsa*I (methylation insensitive) alone as a control and the other with *Rsa*I plus *Hpa*II (methylation sensitive). *Hpa*II is blocked by CpG methylation and digests the active X chromosome solely. The digests were incubated at 80 °C for 20 min to inactivate the enzymes and then used for PCR amplification of a polymorphic (CAG)n repeated in AR. Each 25 μL PCR reaction mixture contains 100 ng genomic DNA, 0.4 mM of each dNTP, 1.5 mM MgCl2, 1x PCR buffer, 20 pmol of each primer (forward: 5′-ACCGAGGAGCTTTCCAGAAT-3′ and reverse: 5′-[Cy5]-CTCATCCAGGACCAGGTAGC-3′), and 0.5 U FastStart Taq DNA polymerase (Roche diagnostics, Manheim, Germany). The PCR was performed on a Veriti™ 96-well Thermal Cycler (Applied Biosystems, Foster City, CA, USA) with a cycling condition of 95 °C for 5 min, followed by 40 cycles of 95 °C for 30 sec, 57 °C for 30 sec, and 71 °C for 3 min, and a final extension cycle at 71 °C for 5 min. Because the PCR primers flank the *Hpa*II sites, after *Hpa*II digestion, only AR alleles in inactivated status were amplified. The degree of XCI was determined based on the ratio of the peak height of each AR allele relative to the sum of the heights of both AR alleles. Skewed XCI was considered if one of the two AR alleles was inactivated for more than 65%. For cases that showed the uninformative result in AR analysis due to the two alleles being identical in amplicon size, a second XCI assay was performed by examination the variable number of tandem repeats in monoamine oxidase A (MAOA) promotor [14]. The testing procedure is identical to AR analysis except that the restriction enzyme *Rsa*I used for DNA digestion is replaced by *HindIII* (methylation insensitive), and the PCR primer set used is of forwarding: 5′-GCGTGCTCCAGAAACATGAG and reverse: 5′-[Cy5]-GCTGTAGGAGGTGTCGTCCA-3′. The XCI pattern was examined on GenomeLab™ GeXP Genetic Analysis System (Beckman Coulter Inc., Brea, CA, USA).

### 2.6. Linkage Analysis

Linkage analysis by short tandem repeat (STR) markers was performed to determine the parental origin of chromosomes and check whether a chromosome rearrangement occurs in patients. Five STR markers located on X chromosome (DXS9901, F8int9.2, F8C-IVS13, F8int21, F8C-IVS22) were selected for this analysis [15,16]. Primer sets and PCR conditions for the STR amplification were summarized in Table S3. The linkage analysis was performed on GenomeLab™ GeXP Genetic Analysis System (Beckman Coulter Inc., CA, USA).

## 3. Results

### 3.1. Cytogenetic Analysis

The cytogenetic analysis identified a normal karyotype in all the individuals who participated in this study: 46,XX for female cases of 1.2, 1.3, 1.4, 2.2, 2.3, 2.4, 3.2, 3.3, and 3.7, and 46,XY for male cases of 1.1 and 2.1.

### 3.2. Molecular Genetic Examinations, XCI Analysis, and Linkage Analysis

Different *F8 or F9* mutations were identified by DNA sequencing, confirming the diagnosis of HA in the female patients of families 1 and 2, and HB in the female patient of family 3 (Figure 1).

In the female patient of family 1 (case 1.3), a heterozygous missense NM_000132.3:c.6683G>A mutation was detected in the exon 24 of the *F8* gene. The mutation led to a substitution of Arginine with Glutamine at amino acid 2228 (p.R2228Q). Genetic testing for IVS22, IVS1, and deletions by inverse PCRs and MLPA was negative. Parental follow-up analysis showed that the c.6683G>A mutation was inherited from her unaffected mother (case 1.2). No *F8* mutation was found in the unaffected father (case 1.1) and younger sister (case 1.4). The XCI assay by AR analysis showed that the female patient (case 1.3) is heterozygous for two alleles with different length sizes: 268 and 282 base pair (bp), respectively; the former is recognized as the maternal allele and the latter is the non-maternal (i.e., paternal) allele (Table 1). After *Rsa*I + *Hpa*II digestion, only the 282-bp allele was amplified, indicating a completely skewed XCI of the paternal allele (100% inactivated) and corresponding to unmethylation (100% activated) of the maternal (mutant) allele. Skewed XCI was also found in the unaffected sister (case 1.4) (XCI ratio of the paternal and maternal allele = 68%: 32%) but not in the mother (case 1.2) (XCI ratio of the two alleles = 50%: 50%) (Table 1). Linkage analysis and XCI analysis confirmed that the female patient (case 1.3) and her unaffected sister (case 1.4) inherited different copies of X chromosomes (one with the *F8* mutation and the other without the *F8* mutation) from their mother (Figure 1 and Table 1). Linkage analysis provided no evidence of X-chromosome recombination in this family (Figure 1).

In the female patient of family 2 (case 2.3), sequencing analysis detected a novel heterozygous nonsense NM_000132.3:c.4814C>A mutation in the exon 14 of the *F8* gene. The mutation changed the amino acid Serine1605 to a stop codon (p.S1605*), resulting in premature termination of translation. The mutation was not detected in her parents, indicating a de novo origin. Further genetic analyses excluded the presence of IVS22, IVS1, and deletions in *F8* gene. XCI assay by AR analysis for the female patient (case 2.3) and her unaffected sister (case 2.4) was uninformative since the two alleles are identical in size (275 bp) in both cases (Table 1). The mother (case 2.2) have two different AR alleles (265 and 275 bp) without skewed XCI (Table 1). A second round of XCI analysis for the MAOA locus was then performed. The mother showed only one-size of allele (242 bp) but the female patient (case 2.3) and her unaffected sister (case 2.4) presented with two different alleles (maternal 242 bp and paternal 271 bp) (Table 1). Skewed XCI of the maternal (242-bp) allele was identified in the female patient (80% in case 2.3) and her unaffected sister (86% in case 2.4), corresponding to a hypomethylation (i.e., highly activated) of the paternal allele (Table 1). The father refused to participate in the linkage experiment, but linkage results from the mother and daughters suggested no X-chromosome recombination in this family (Figure 1).

In the female patient of family 3 (case 3.7), a heterozygous missense NM_000133.3:c.532T>C mutation was detected in the exon 6 of the *F9* gene. The mutation led to a substitution of Cysteine with Arginine at amino acid 178 (p.C178R). The mutation was not detected in the mother (case 3.2), and the late father showed no hemophilia history when he was alive, suggesting a de novo origin. The XCI assay by AR analysis identified two different alleles in the female patient (case 3.7) (275 bp and 279 bp) and her unaffected sister (case 3.3) (275 bp and 279 bp) (Table 1). The 275-bp allele detected in both sisters was not found in the mother (case 3.2), suggesting a paternal origin (Table 1). The 279-bp allele that can be found in the mother (case 3.2) was recognized as the maternal copy. Linkage analysis showed that the female patient (case 3.7) and her sister (case 1.3) inherited an identical copy of X chromosome from their mother (Figure 1), consistent with the finding of the XCI analysis (Table 1). The maternal allele of the patient (case 3.7) is totally skewed XCI (100% inactivated), indicating unmethylation (100% activated) of the paternal allele (Table 1). Neither the mother (case 3.2) nor the elder sister (case 3.3) showed XCI (50%: 50% in both cases). Linkage analysis provided no evidence of X-chromosome recombination in this family (Figure 1).

## 4. Discussion

Female carriers of heterozygous mutations theoretically do not present with X-linked recessive hemophilias because of a backup of one normal copy of the X chromosome. However, symptomatic female carriers are found in several X-linked recessive disorders and a number of mechanisms have been proposed to explain this observation [17,18]. Skewed XCI is one of the most frequently reported mechanisms, by which the majority of active X-chromosome carries the mutant allele. In this study, all the manifesting female patients (cases 1.3, 2.3, and 3.7) were demonstrated to carry only one heterozygous mutation in the causative genes (*F8* and *F9*) and share the mechanism of skewed XCI.

XCI (also known Lyonization) is the process of permanently inactivating one of the two X chromosomes in females and it happens from a series of essentially irreversible chemical modifications to silence genes on the inactive X chromosome [19]. The inactivation equalizes gene expression of the X chromosome between XX females and XY males. The inactivated X chromosome is randomly selected. Therefore, females are generally mosaic, that is, some cells activate the paternal X chromosome and others activate the maternal X chromosome. Skewed XCI occurs when one X chromosome is prone to be inactivated that leads to unequal ratio of cells with each chromosome inactivated.

In family 1, the female patient (case 1.3) inherited a heterozygous *F8* mutation from her mother and exhibited extremely skewed (100%) XCI of the paternal/wild-type allele. The complete activation of the maternal/mutant copy of *F8* possibly altered the phenotype from “unaffected” to “affected”, providing a reasonable explanation for the clinical manifestation of the patient. On the contrary, the patient’s mother (case 1.2) showed equal XCI in both X chromosomes (50:50) and being a non-symptomatic carrier; it is interesting to note that the patient’s sister (case 1.4) did not inherit the maternal mutation but also showed skewed (68%) XCI in the paternal X chromosome; this observation suggests that the skewed XCI pattern is not inherited and is variable in this generation of family 1.

In family 2, the female patient (case 2.3) is a sporadic case with a de novo mutation in *F8* gene. Skewed (80%) XCI of the maternal X chromosome found in this case indicates that the majority of *F8* expression is the paternal allele. De novo mutations account for about 33% of all cases of HA. However, as with all genetic disorders, de novo mutations can occur post-zygotically, or pre-zygotically [20,21,22]. In the former scenario, mutations arise in the first few cell divisions after fertilization that leads to high-level mosaicism and are present in many different tissues. In the latter situation, mutations preexisted in a parent of unrevealed mosaicism (gonadal mosaicism) that can be transmitted to the next generation and cause diseases [20,21,22]. The *F8* sequencing using DNA from the peripheral blood cells of the patient (case 2.3) showed no evidence of mosaicism for the de novo mutation, suggesting paternal gonadal mosaicism, followed by skewed XCI, contributes to the manifestation of hemophilia in the patient. Gonadal mosaicism is a phenomenon difficult to be identified but has be implicated in a number of studies. For example, it has been shown the recurrence rate for a couple that has a child with a genetic disease caused by a de novo variant is 1–4% higher than that of the general population [21,23]. Notably, the patient’s sister (case 2.4) also showed skewed (86%) XCI of the maternal X chromosome but is phenotypically normal; it is possible that the patient’s sister inherited a X chromosome with a wide-type allele from her father in whom gonadal mosaicism existed.

In family 3, the female patient (case 3.7) is a sporadic case with a de novo mutation in *F9* gene. De novo mutations were reported in about 30% of HB cases. The extremely skewed (100%) XCI of the maternal X chromosome found in this case indicates that the *F8* expression is the paternal allele. The patient’s father was died but he showed no hemophilia history when he was alive. Therefore, paternal gonadal mosaicism, as suggested in family 2, may also contribute to the molecular pathology of the patient (case 3.7). However, true mosaicism (gonadal mosaicism plus somatic mosaicism) cannot be completely excluded from the patient’s father because genetic testing for the father is infeasible. True mosaicisms frequently lead to a range of clinical phenotypes, depending on the population of cells affected [24]. Normal phenotype (asymptomatic) in the patient’s father may be attributed to the presence of a sufficient proportion of cells with a normal allele in tissues [25].

Significant deviation from the random pattern of XCI is a hallmark for heterozygous females with severe X-linked diseases [13]. Differential methylation analysis at the AR gene is commonly used in the XCI analysis. The major advantage of AR gene for XCI analysis is the high heterozygosity of the CAG repeat polymorphism in exon 1 of AR. The two X chromosomes of females cannot be distinguished by AR analysis is estimated to be 10% [26]. However, noninformative XCI results still may be obtained by the AR analysis, as shown in our cases 2.3 and 2.4 of family 2. To cope with this plight, additional loci can be considered for further XCI analyses. A number of X-linked loci feasible for XCI analyses has been reported that include MAOA, FMR1, GRIA3, PGK1, ZNF261, ZDHHC15, SLITRK4, and PCSK1N [27,28,29,30,31]. The estimated frequencies of heterozygosity for these loci vary, for example, 58% for *ZDHHC15*, 80% for *SLITRK4*, and 75% for *PCSKIN* [31]. In our family 2, a second round of XCI analysis performed with MAOA locus provided informative results for cases 2.3 and 2.4.

Apart from skewed XCI, other mechanisms have been documented in females with X-linked hemophilia. For example, when the coexistence with numerical, or structural X-chromosome abnormalities (e.g., Turner syndrome, translocations), it would lead to affected females by interfering the gene expression [2,32,33]. In addition, some exceptional examples may also cause symptomatic females (e.g., homozygous mutation resulted from consanguineous marriage) [3]. Moreover, a 46,XY female as a result of a mutation in the sex-determining region of the Y-chromosome (*SRY*) gene on the Y chromosome combined with a mutation in the *F8* gene was also reported as an exceptional example causing phenotypic heterozygous female [34]. Some reported female HA cases turned out to be a misdiagnosis, since they were actually von Willebrand disease (VWD) type 2N that resulted in decreased FVIII bleeding capacity [35].

Although hemophilias are well-documented X-linked recessive bleeding disorders, female patients are relatively rare and mechanisms involved in the molecular pathology are complex and much uncertain. Therefore, in addition to conventional assays to analyze coagulation function, a combination of genetic analyses (e.g., chromosomal complement, molecular genetic examinations (e.g., mutational screening of the causative genes), XCI assay, linkage analysis) is required to explicit the underlying mechanism in female hemophilia, especially when only one mutation is detected. Notably, a recent study for heterozygous female HA patients indicated that the main reason for skewed XCI in patients was negative selection against cells with a disadvantage caused by an additional deleterious mutation in hemophilia-unrelated genes on the silenced X chromosome (i.e., the X chromosome without the HA mutation) [36]. If the hypothesis is true, the two X chromosomes of affected females will be expected to be either homozygous or compound heterozygous for hemophilia mutations, or have a hemophilia mutation in one X chromosome combined with a deleterious mutation in hemophilia-unrelated genes on the other X chromosome that will render preimplantation genetic testing infeasible. Oocyte donation is therefore a reasonable choice to offer during genetic counseling. To test the hypothesis, incorporating whole exome sequencing (WES) into our further test panel for female hemophilia patients with skewed XCI is considered justified nowadays in our department.

## 5. Conclusions

Skewed XCI is confirmed as a common mechanism responsible for HA and HB in females heterozygous for a single mutation. For female patients with a de novo mutation, parental germline mosaicism, followed by skewed XCI, may contribute to hemophilia. Since the complex nature of mechanisms responsible for hemophilia as well as other X-linked recessive disorders in female patients, a combination of genetic analyses, in which more loci for XCI analysis may be included, is required.

## Figures and Tables

**Table 1:** The X-chromosome inactivation (XCI) patterns in female members of families 1, 2, and 3. Skewed XCI is evidenced in the three patients (case 1.3. 2.3, and 3.7) and two unaffected females (case 1.4 and 2.4), all showed unequal methylated degree between the two alleles of androgen receptor (AR), or monoamine oxidase A (MAOA) locus. Nonmethylation of the maternal allele was demonstrated in one patient (case 1.3: 0%). Hypomethylation, or nonmethylation of the paternal allele was evidenced in two patients (case 2.3: 20% and case 3.7: 0%).

	AR XCI Ratio		MAOA XCI Ratio	
	Allele 1 ^†^	Allele 2 ^†^	Allele 1 ^†^	Allele 2 ^†^
**Family 1**				
case 1.2 *	50% (278)	50% (268)	NP	NP
case 1.3 *	100% (282)	0 (268)	NP	NP
case 1.4 *	68% (282)	32% (278)	NP	NP
**Family 2**				
case 2.2 *	50% (265)	50% (275)	Uninformative ^††^ (242)	Uninformative ^††^ (242)
case 2.3 *	Uninformative ^††^ (275)	Uninformative ^††^ (275)	20% (271)	80% (242)
case 2.4 *	Uninformative ^††^ (275)	Uninformative ^††^ (275)	14% (271)	86% (242)
**Family 3**				
case 3.2 *	50% (285)	50% (279)	NP	NP
case 3.3 *	50% (275)	50% (279)	NP	NP
case 3.7 *	0% (275)	100% (279)	NP	NP

* Case numbers are the same as in Figure 1. ^†^ Numbers in the parentheses indicate the allele sizes (base pairs) detected in XCI analyses with AR, or MAOA. ^††^ The XCI ratios of alleles cannot be determined because the two alleles (alleles 1 and 2) are identical in amplicon size. NP, not performed.

## Data Availability

Data are available upon request.

## Supplementary Materials

The following supporting information can be downloaded at: https://www.mdpi.com/article/10.3390/diagnostics12102267/s1, Table S1: Primer sets used for polymerase chain reaction (PCR) amplification of the 26 exons of human *F8* gene; Table S2: Primer sets used for PCR amplification of the 8 exons of human *F9* gene; Table S3: Primer sets for PCR amplification of five short tandem repeat (STR) markers on X chromosome in linkage analysis.
