# Supplementary material for: Skewed X-Chromosome Inactivation and Parental Gonadal Mosaicism Are Implicated in X-Linked Recessive Female Hemophilia Patients
Source: Diagnostics (Basel). 2022 Sep 20;12(10):2267. doi: 10.3390/diagnostics12102267 (PMC9600608; doi:10.3390/diagnostics12102267)
Supplement: Supplementary file 1 [file diagnostics-12-02267-s001.zip › TabS3_STR primers.pdf]

Table S3. Primer sets for PCR amplification of five short tandem repeat (STR) markers on X chromosome in linkage analysis.

STR marker	Primer set*
**DXS9901**	DXS9901-F: GACCAGTCCTCCCTTCTGTT<br>DXS9901-R: GTGTGGAGTGAAAGGGACAG
**F8int9.2**	F8int9.2-F: ATCACACCACAGCACTCCAG<br>F81nt9.2-R: TGCAGCTTCCATGTTATTGC
**F8C-IVS13**	F8C-IVS13-F: TGCATTCAACTGTACATAATGTATCTT<br>F8C-IVS13-R: CCAAATTACAGATTGAATAAGCCTAG
**F8int21**	F8int21-F: AGCCTGCTTCTTGACAAACC<br>F8int21-R: GTTTCAGGAGAGGGGAGGAA
**F8C-IVS22**	F8C-IVS22-F: ATTAATGCCCACATTATAGACTCTC<br>F8C-IVS22-R: AATAAGACCCTTAGCTGTTTCAT

*F, forward; R, reverse.

All PCRs were performed with the same condition: 95°C, 5 min → 96°C, 2 min → (94°C, 1 min → 56°C, 1 min → 70°C, 1 min)$_{27}$ → 60°C, 30 min.
