# Supplementary material for: Skewed X-Chromosome Inactivation and Parental Gonadal Mosaicism Are Implicated in X-Linked Recessive Female Hemophilia Patients
Source: Diagnostics (Basel). 2022 Sep 20;12(10):2267. doi: 10.3390/diagnostics12102267 (PMC9600608; doi:10.3390/diagnostics12102267)
Supplement: Supplementary file 1 [file diagnostics-12-02267-s001.zip › TabS1_F8 primers and PCR condition.pdf]

Table S1. Primer sets used for polymerase chain reaction (PCR) amplification of the 26 exons of human *F8* gene.

Exon	Primer set*	Fragment size (bp)	Exon	Primer set*	Fragment size (bp)
**1**	F8-1-F1:GTTTAGCAGCCTCCCTTTTG<br>F8-1-R1:AGATGTGTGCACACCTTACC	544	**14-G**	F8-14 -F7:GGGACAAGTGCCACAAATTC<br>F8-14 -R7:GGGGCTCTGATTTTCATCCT	678
**2**	F8-2-F1:GGCACCTAATGTATCAGTTG<br>F8-2-R1:AACATTCTCTTTGGCAGCTG	417	**14-H**	F8-14 -F8:GCTCTCAAAACCCACCAGTC<br>F8-14 -R8:CATGAAAACCAATCCTCCCC	586
**3**	F8-3-F1:TTCTCCACTGTGACCTTGAC<br>F8-3-R1:ATGACAGGACAATAGGAGGG	249	**15**	F8-15-F1:AATGCTTCTCAGGCACCTAG<br>F8-15-R1:TCCACTGTCCTTAACTCACC	268
**4**	F8-4-F1:TCTTCCTGCTATAGGAGCTG<br>F8-4-R1:CTACATACTAGTAGGGCTCC	222	**16**	F8-16-F1:TTTTTTTTTTGTCGTTATTGTTCTAC<br>F8-16-R1:TCAGCACAATAGACACCTGCTT	265
**5**	F8-5-F1:GATGAAGAACTGTCTCCTCC<br>F8-5-R1:AACCCCATCTCCTTCATTCC	274	**17**	F8-17-F1:GCAGGTTGGACTGGCATAAA<br>F8-17-R1:GAGGATTCCACTCCCACAGA	447
**6**	F8-6-F1:CTACGATTACAGGTGTGAGC<br>F8-6-R1:GTACAGAACTCTGGTGCTGA	563	**18**	F8-18-F1:TTGGTGGAGTGGAGAGAAAG<br>F8-18-R1:ACTGATTGTGTTCCCAGTGC	338
**7**	F8-7-F1:TACAGGTCTGATTGGATGCC<br>F8-7-R1:TACCATGTTGGTGGGAAGAG	230	**19**	F8-19-F1:TGAGGGGTCCAAAAGTAGTG<br>F8-19-R1:ACCTCTGCCCACATTGCTAC	270
**8**	F8-8-F1:AATTTCCAATGCTGGAGGTG<br>F8-8-R1:GCAACTGAGCGAATTTGGAT	532	**20**	F8-20-F1:GTTGACGTTCTCCCATTTTC<br>F8-20-R1:TAATCAGCCCAGGTTCTTGG	202
**9**	F8-9-F1:CAGAGGAACCCCAACTACGA<br>F8-9-R1:ATGTCCATTGGAGACAAGGC	783	**21**	F8-21-F1:TACTTGGGCAAAGGACAGTG<br>F8-21-R1:TTGAGCTTGCAAGAGGAATAAG	175
**10**	F8-10-F1:GACTTGAGCATCACAGATTG<br>F8-10-R1:TCCTTGAATACAAAGGACGG	308	**22**	F8-22-F1:ATTGGTAGGTGGATCTGTTG<br>F8-22-R1:TACCATTAAGGTTCCAGTGG	167
**11**	F8-11-F1:GTTTTGCTTGTGGGTAGGTG<br>F8-11-R1:GGGGACATACACTGAGAATG	283	**23**	F8-23-F1:TGTTCTTGAGCTCTACCAGG<br>F8-23-R1:TTTTGTGTCCTGATACCGGG	771
**12**	F8-12-F1:TGCCATCGCTTTCATCATAG<br>F8-12-R1:GGGTTATATGATCACGTGTG	402	**24**	F8-24-F1:GCTGCTCAGTATAACTGAGG<br>F8-24-R1:TCTGTTGCCTCTTACCTGAG	229
**13**	F8-13-F1:CTGGGAATAAGATAATGGGC<br>F8-13-R1:ACTAACCTGGGTTTTCCATC	333	**25**	F8-25-F1:AGGGATTTGGGAATTTCTGG<br>F8-25-R1:CAGCTTACCTTTACTTTGCC	253
**14-A**	F8-14 -F1:ATTCTGCAAGTGGGTGACAG<br>F8-14 -R1:GCCTCTGTTCCGAAAGTCTG	635	**26-A**	F8-26-F1:TCACTGAGGTTTGGGAAGTG<br>F8-26-R1:GCTTTCATGCAGGTTTCTCC	682
**14-B**	F8-14 -F2:CCAGGGATTTAACCCAATGA<br>F8-14 -R2:GTGTCATTTCAGACAGGCTG	593	**26-B**	F8-26-F2:TGGCTTGCCTTCTACCTTTG<br>F8-26-R2:CTTAGGGATTCTTGAACCCC	610
**14-C**	F8-14 -F3:TCTGATGATCCATCACCTGG<br>F8-14 -R3:ATGGGCCATCAATGTGAGTC	607	**26-C**	F8-26-F3:AGGGGCACATTCTTATCTCC<br>F8-26-R3:TGGTGATATGGCAGACTGGA	560
**14-D**	F8-14 -F4:AGCTCATGGACCTGCTTTGT<br>F8-14 -R4:CCATCTCTTTGAGTCCTACG	569	**26-D**	F8-26-F4:GGTCAGAAGAAAATTGGACTGG<br>F8-26-R4:TCTTGCTATTCAGTGCCCCTA	787

**14-E**	F8-14 -F5:AGTGGTAGTAGGAAAGGGTG<br>F8-14 -R5:GCCCCTTTCTCCTTCTCATT	645	**26-E**	F8-26-F5:GGCATTCTTTTCCCATTGAC<br>F8-26-R5:ATCTTGGCTCACTGCAACCT	649
**14-F**	F8-14 -F6:CAGAATTTTGTCACGCAACG<br>F8-14 -R6:GATGTTTTGGGCAAGTCTGG	557			

*F, forward; R, reverse.

All PCRs were performed with the same condition: 95°C, 5 min → (95°C, 45 sec → 55°C, 45 sec → 71°C, 45 sec)$_{40}$ → 71°C, 1 min → 4°C, 1 min.
