# Supplementary material for: Skewed X-Chromosome Inactivation and Parental Gonadal Mosaicism Are Implicated in X-Linked Recessive Female Hemophilia Patients
Source: Diagnostics (Basel). 2022 Sep 20;12(10):2267. doi: 10.3390/diagnostics12102267 (PMC9600608; doi:10.3390/diagnostics12102267)
Supplement: Supplementary file 1 [file diagnostics-12-02267-s001.zip › TabS2_F9 primers and PCR condition.pdf]

**Table S2.** Primer sets used for PCR amplification of the 8 exons of human *F9* gene.

| Exon | Primer set* | Fragment size (bp) | Exon | Primer set* | Fragment size (bp) |
|---|---|---|---|---|---|
| **1** | F9-1-F1:AGTCCAAAGACCCATTGAGG<br>F9-1-R1:GACTCTTCAATATTGCTGTCAAATC | 321 | **6** | F9-6-F1:TACTGATGGGCCTGCTTCTC<br>F9-6-R1:AATAGCCTCAGTCTCCCACCT | 398 |
| **2** | F9-2-F1:TGCCCTAAAGAGAAATTGGC<br>F9-2-R1:TGCTCTGCATCTGAAGGGTAT | 327 | **7** | F9-7-F1:GCCTATTCCTGTAACCAGCAC<br>F9-7-R1:GCCTTTAGCCCAATTTATTCA | 315 |
| **3** | F9-3-F1:CCCTTCAGATGCAGAGCATAG<br>F9-3-R1:GGTTGGACTGATCTTTCTGAGTC | 318 | **8-A** | F9-8-F1:GCCAATTAGGTCAGTGGTCC<br>F9-8-R1:CTTCATGGAAGCCAGCAC | 458 |
| **4** | F9-4-F1:GCTGGCTTCCAGGTCAGTAG<br>F9-4-R1:TCAGAGGGAAACTTTGAACCA | 291 | **8-B** | F9-8-F2:TGTAAGTGGCTGGGGAAGAG<br>F9-8-R2:TGAGAGGCCCTGTTAATTTTC | 388 |
| **5** | F9-5-F1:CCCCAATGTATATTTGACCCATAC<br>F9-5-R1:CAAAAGGAAGCAGATTCAAGTAGG | 333 | | | |

*F, forward; R, reverse.

All PCRs were performed with the same condition: 95°C, 5 min → (95°C, 40 sec → 55°C, 40 sec → 71°C, 45 sec)$_{40}$ → 71°C, 1 min → 4°C, 1 min.
